# Predictive Modeling of Central Precocious Puberty Using IGF-1 and IGFBP-3 Standard Deviation Scores

**DOI:** 10.3390/diagnostics15192508

**Published:** 2025-10-02

**Authors:** Rihwa Choi, Gayoung Chun, Sung-Eun Cho, Sang Gon Lee

**Affiliations:** 1 Laboratory Medicine Center, Division of Laboratory Medicine, GC Labs, Yongin-si 16924, Republic of Korea; pirate0720@naver.com; 2 Department of Laboratory Medicine and Genetics, Sungkyunkwan University School of Medicine, Seoul 06351, Republic of Korea; 3 Biostatistics Team, Infectious Disease Research Center, Division of Laboratory Medicine, GC Labs, Yongin-si 16924, Republic of Korea; forjund@gclabs.co.kr; 4 Endocrine Substance Analysis Center (ESAC), Division of Laboratory Medicine, GC Labs, Yongin-si 16924, Republic of Korea

**Keywords:** central precocious puberty, insulin-like growth factor I, insulin-like growth factor-binding protein 3, luteinizing hormone, follicle-stimulating hormone, standard deviation scores

## Abstract

**Background/Objectives**: Central precocious puberty (CPP) is diagnosed via gonadotropin-releasing hormone (GnRH) stimulation testing, which can be burdensome in pediatric settings. This study evaluated the utility of baseline hormonal markers—particularly insulin-like growth fac-tor 1 (IGF-1) and IGF-binding protein 3 (IGFBP-3)—as auxiliary tools for CPP diagnosis in Korean children. **Methods**: We retrospectively analyzed patients who underwent GnRH stimulation testing. Baseline LH, FSH, IGF-1, and IGFBP-3 levels were assessed, along with standard deviation scores (SDS) calculated using two different reference intervals. Multivariable logistic regression was performed to improve diagnostic accuracy. Performance was evaluated using area under the curve (AUC) values from receiver operating characteristic (ROC) analyses, stratified by sex. **Results**: Among 2464 Korean children (2025 girls and 439 boys), CPP diagnosis rates were 54.2% in girls and 65.6% in boys. Among baseline markers, FSH showed the highest AUCs using raw values with sex-specific cutoffs (AUC = 0.767 in girls and 0.895 in boys). Although IGF-1 SDS and IGFBP-3 SDS showed AUCs < 0.7 when used alone, predictive models incorporating these SDS values yielded higher performance (AUC = 0.800 in girls and 0.920 in boys. **Conclusions**: SDS-based IGF-1 and IGFBP-3 enhance CPP diagnosis when used in predictive models, emphasizing the need for sex-specific interpretation and standardized reference intervals in real-world clinical practice.

## 1. Introduction

Precocious puberty is defined as the onset of secondary sexual characteristics before the age of 8 years in girls and 9 years in boys, due to premature activation of the hypothalamic–pituitary–gonadal (HPG) axis, resulting in central precocious puberty (CPP) [1,2]. Diagnosis of CPP typically involves assessment of physical signs of puberty, advancement of bone age, and confirmation by a Gonadotropin-releasing hormone (GnRH) stimulation test demonstrating a pubertal luteinizing hormone (LH) response [1,2]. In Korea, treatment with GnRH agonists (such as goserelin, leuprolide, or triptorelin) is indicated in girls under the age of 7 years and 365 days and boys under the age of 8 years and 365 days who present with secondary sexual characteristics, advanced bone age relative to chronological age, and a peak LH level of ≥5 IU/L with a 2–3-fold increase from baseline following a GnRH stimulation test [3]. Furthermore, if GnRH agonist therapy is initiated before 8 years and 365 days in girls or before 9 years and 365 days in boys, the cost of treatment is reimbursed under the national health insurance system until the age of 11 years and 364 days for girls and 12 years and 364 days for boys [3,4]. The GnRH stimulation test, which is the definitive diagnostic test for CPP, requires multiple serial blood samples after pharmacologic stimulation [2]. Due to the invasive nature of the procedure, it is often considered challenging to perform in pediatric populations. Consequently, several auxiliary biomarkers—such as baseline LH, baseline LH/follicle-stimulating hormone (FSH) ratio, Insulin-like growth factor-1 (IGF-1), insulin-like growth factor binding protein-3 (IGFBP-3), and the IGF-1/IGFBP-3 ratio—have been investigated as potential tools to aid clinical decision-making prior to confirmatory testing [2,4,5]. The 2022 Korean guidelines for the diagnosis of CPP suggest that baseline LH may serve as an auxiliary marker in the diagnostic process [2]. However, the optimal cutoff values for basal LH remain a subject of debate [1,2].

IGF-1 and IGFBP-3 have been suggested as biomarkers that reflect GH axis activity and pubertal development [1,4,5,6,7]. These biomarkers are relatively stable compared to pulsatile gonadotropins and show age- and sex-dependent increases during puberty [1]. Several studies have suggested their potential roles as adjuncts in the evaluation of CPP, in supporting or prioritizing GnRH stimulation testing in early or borderline cases [1,6,7].

LH, FSH, IGF-1, and IGFBP-3 levels are measured using immunoassay-based techniques [1,2,8,9,10]. However, poor inter-assay standardization and differences in antibody specificity, calibration materials, and matrix effects have led to substantial variability in reference values across platforms and populations [6,7,8,9,10,11,12,13,14,15,16]. Because antigen–antibody binding reactions vary by manufacturer, the presence of genetic variations at the binding site may lead to discrepant test results depending on the specific assay used [1,16]. Consequently, clinical interpretation requires population-specific reference ranges and assay-specific cutoffs [1]. These differences in cutoffs can affect the interpretation of IGF-1 and IGFBP-3—such as the application of standard deviation scores (SDS) values—and may contribute to inconsistencies in studies evaluating the performance of IGF-1 and IGFBP-3 as adjunctive markers in the diagnosis of CPP [1,10,11,12,13,14,15,16,17,18].

Recently, Roche Diagnostics has developed fully automated electrochemiluminescence immunoassays (ECLIAs) for IGF-1 and IGFBP-3, traceable to the WHO International Standard (02/254 for IGF-1) and liquid chromatography tandem mass spectrometry-based reference procedures [1,10,13,19]. According to the manufacturer Roche’s Instructions for Use, IGFBP-3 is standardized against the IDS iSYS method, which in turn is traceable to the NIBSC International Standard 93/560 [10]. Despite these standardization efforts, substantial discrepancies still exist between different assay methods, and reference data for pediatric and adolescent populations remain limited [1,9,10]. To date, only one population-based study in Korean children has reported age- and sex-specific reference ranges using this Roche platform, while the manufacturer also provides expected values based on a multicenter cohort [10]. Therefore, it remains unknown whether IGF-1 and IGFBP-3 measured using the Roche platform can aid in the diagnosis of CPP in Korean children and adolescents.

Given this background, the present study aimed to evaluate the diagnostic performance of IGF-1 and IGFBP-3 concentrations in differentiating CPP from non-CPP in Korean children. We assessed the clinical utility of these markers using both manufacturer-provided and Korean population-derived reference intervals [10].

## 2. Materials and Methods

### 2.1. Study Population

We retrospectively reviewed the laboratory data of pediatric patients who underwent GnRH stimulation testing between 10 February 2022 and 9 August 2023. This study included laboratory results from children who presented to local pediatric clinics or hospitals across South Korea and were referred to GC Labs for simultaneous assessment of serum TSH, LH, FSH, IGF-1, and IGFBP-3 levels. GC Labs is a nationwide clinical laboratory that provides comprehensive diagnostic services, including hormone assays [20]. The following exclusion criteria were applied: 1. Age at the time of the GnRH stimulation test exceeding 8 years and 365 days in girls or before 9 years and 365 days in boys; 2. Missing baseline measurements for any of the following: serum TSH, LH, FSH, IGF-1, or IGFBP-3; 3. Serum TSH levels outside the predefined reference range of 0.59–7.39 μIU/mL [21]. Missing data were addressed through exclusion: two individuals without simultaneous baseline LH and FSH measurements were excluded during initial eligibility screening.

The study was conducted in accordance with the Declaration of Helsinki, and approved by the Institutional Review Board (IRB) of GC Labs (GCL 2024-1056-01, 10 September 2024). A waiver of informed consent was approved by the IRB as this study was retrospective and involved no more than minimal risk to subjects (GCL 2024-1056-01, 10 September 2024).

### 2.2. Analytical Methods

Serum TSH, LH, FSH, IGF-1, and IGFBP-3 were measured using Elecsys reagents on the cobas 8000 e801 analyzer (Roche, Mannheim, Germany). All assays were performed in accordance with the manufacturer’s instructions, using automated ECLIA technology [10].

### 2.3. Definitions

CPP was defined based on the Korean diagnostic and reimbursement criteria, as undergoing a GnRH stimulation test before the chronological age of 8 years and 365 days in girls or before 9 years and 365 days in boys [2,3]. A positive test result was defined as a peak LH concentration ≥ 5 IU/L with a 2- to 3-fold increase from baseline following administration of GnRH [2,3]. Given the retrospective design, potential sources of misclassification and information bias were mitigated by applying standardized inclusion and exclusion criteria, using only hormone tests performed by a single central laboratory (GC Labs), and relying on electronic laboratory records that consistently recorded baseline hormone levels and diagnosis information. Additionally, interpretation of CPP diagnosis was based on uniform biochemical criteria, reducing the risk of subjective misclassification.

### 2.4. Statistical Methods

Continuous variables that were not normally distributed were presented as medians and interquartile ranges (IQRs). Categorical variables were expressed as counts and percentages (*n*, %). To analyze LH test results below the lower limit of quantification (LLOQ; < 0.3 IU/L), values were imputed by dividing the LLOQ by the square root of 2 (i.e., LH = 0.3/√2) [22]. Considering the sex-specific differences in the development of the hypothalamic–pituitary–gonadal axis, statistical analyses were performed separately for boys and girls [1,2,10].

Although unstimulated LH measurement is considered an auxiliary method for diagnosing CPP according to the 2022 Korean guidelines, the cutoffs for estimating pubertal status have not been widely validated in the Korean population using the Roche assay [2]. Therefore, two baseline LH cutoffs were evaluated: >0.3 IU/L, corresponding to the lower limit of quantification for the Roche method, and >1.1 IU/L, based on a previous study using a radioimmunoassay method [2,23]. In this study, we evaluated the diagnostic performance (sensitivity, specificity, positive predictive value, and negative predictive value) for CPP under the assumption that, in clinical practice, fixed cutoffs for LH and FSH are used without considering assay non-standardization, and that high IGF-1 and IGFBP-3 levels are determined based on the 97.5th percentile of the reference interval without SDS adjustment, as SDS values are often not reported by clinical laboratories.

The SDS for IGF-1 and IGFBP-3 were calculated using the lambda-mu-sigma (LMS) method [10,24]. The SDS was derived using the formula (X − μ)/σ, where μ was set as the median and σ was estimated as the standard deviation based on the approximation that the 97.5th percentile corresponds to the mean plus two standard deviations. When calculated using the reference data provided in the manufacturer’s IFU from Roche, the values were designated as SDS-Roche. When based on the reference intervals established by Jo et al. using the Roche assay, the values were designated as SDS-Jo et al. [10].

To evaluate whether baseline LH, baseline FSH, baseline LH/FSH ratio, IGF-1, IGFBP-3, and IGF-1/IGF-BP3 ratios are useful in diagnosing CPP, receiver operating characteristic (ROC) curve analyses were conducted for each sex. The area under the curve (AUC) was calculated to quantify the overall discriminative ability of baseline LH, baseline LH/FSH ratio, IGF-1, IGFBP-3, and IGF-1/IGF-BP3 ratios in identifying CPP [25].

Multivariable logistic regression models were constructed to assess the combined predictive value of age, sex, baseline LH, baseline FSH, IGF-1 SDS, and IGFBP-3 SDS for CPP diagnosis. Model performance was evaluated using AUCs, and ROC curves were compared using nonparametric DeLong’s test. A *p*-value of less than 0.05 was deemed statistically significant. All analyses were performed using the MedCalc statistical software version 23.2.7 (MedCalc Software Ltd., Ostend, Belgium). Generative artificial intelligence (ChatGPT o3) was used to illustrate the predictive score distributions by sex and CPP diagnostic group. The authors reviewed and validated all AI-assisted outputs and accept full responsibility for the integrity and accuracy of the results presented.

## 3. Results

A total of 3395 pediatric patients were initially screened. After excluding 931 patients who did not meet the age criteria, did not undergo simultaneous measurement of IGF-1 and IGFBP-3, or had serum TSH levels outside the predefined reference range (0.59–7.39 μIU/mL), 2464 children (2025 girls and 439 boys) were included in the final analysis. The median chronological age was 8.1 years (IQR 7.6–8.8) in girls and 9.0 years (IQR 8.6–9.8) in boys. The proportion of patients diagnosed with CPP based on the GnRH stimulation test was 54.2% among girls (1098 subjects) and 65.6% among boys (288 subjects). The baseline characteristics of the study subjects are presented in Table 1.

In the analysis of factors associated with CPP, TSH, IGFBP-3, IGFBP-3 SDS-Roche, and IGFBP-3 SDS-Jo et al. did not show statistically significant differences between the CPP-positive and CPP-negative groups in either sex. In contrast, all other factors showed statistically significant differences between the two groups in both boys and girls (*p* < 0.05, Table 2).

Table 3 summarizes the diagnostic performance (sensitivity, specificity, positive predictive value [PPV], and negative predictive value [NPV]) for CPP when fixed cutoffs for LH and FSH or upper reference limits for IGF-1 and IGFBP-3 are used without accounting for assay standardization or SDS. Baseline LH demonstrated high specificity (>90%) at both cutoffs (>0.3 IU/L and >1.1 IU/L). For baseline FSH, when evaluated using the sex-specific cutoffs that yielded the best AUCs (>1.8 IU/L for girls and >1.6 IU/L for boys), boys showed a high PPV of 91.2%, whereas the PPV in girls was lower at 70.9%. When examining the frequency of IGF-1 and IGFBP-3 levels exceeding the 97.5th percentile values proposed by Roche and Jo et al. in healthy children and adolescents, only elevated IGF-1 levels in girls were significantly more frequent in the CPP-positive group (14.3% based on Roche value and 21.1% based on Jo et al.’s value, respectively) compared to the CPP-negative group 6.4% based on Roche value and 12.8% based on Jo et al.’s value, respectively, *p* < 0.05). No significant differences were observed for the other comparisons (Table 3).

**Figure 1:**
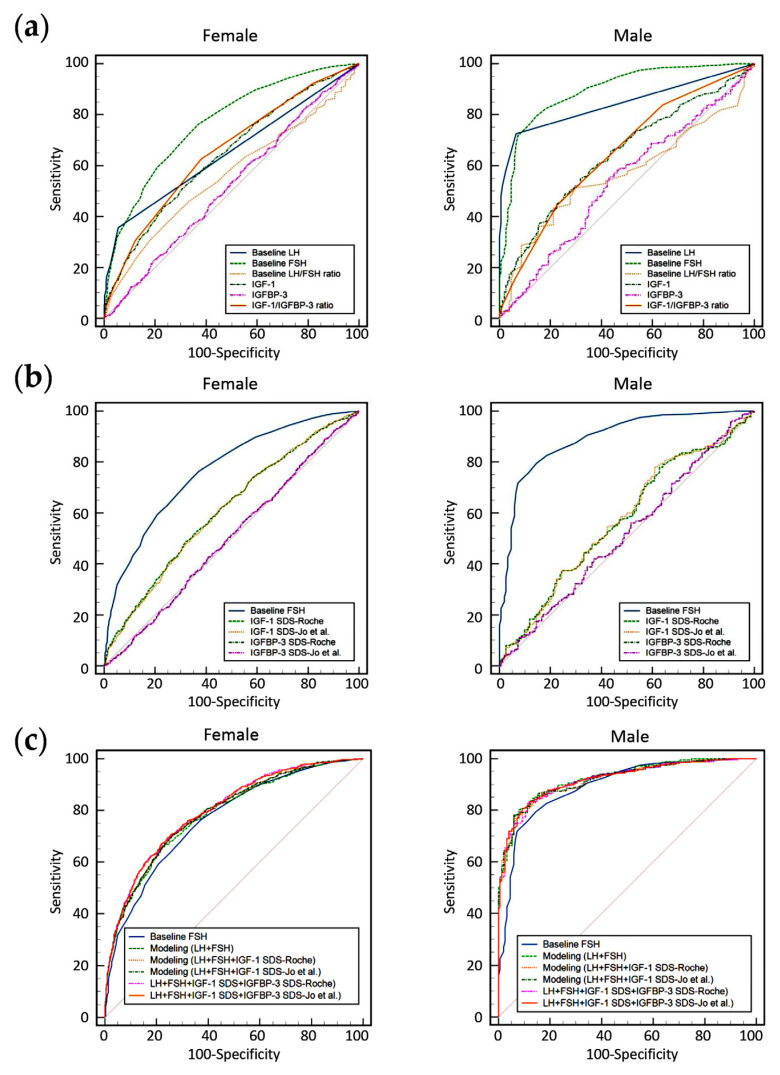
Receiver operating characteristic (ROC) curves for predicting central precocious puberty (CPP), stratified by sex (left: female; right: male). (**a**) ROC curves based on raw values of baseline LH, baseline FSH, baseline LH/FSH ratio, IGF-1, IGFBP-3, and the IGF-1/IGFBP-3 ratio. (**b**) ROC curves based on SDS values for IGF-1 and IGFBP-3, calculated using two different reference interval sources (Roche manufacturer’s instructions for use and Jo et al.), compared with baseline FSH. (**c**) ROC curves for sex-specific predictive models derived from multivariable logistic regression incorporating age, sex, baseline LH, baseline FSH, and SDS values of IGF-1 and IGFBP-3, using reference intervals provided by Roche and the study by Jo et al. [10], compared with baseline FSH alone.

The diagnostic performance of each marker for CPP was evaluated using ROC curve analysis (Figure 1 and Table 4). When using raw measurement values, only baseline FSH in girls and both baseline LH and FSH in boys had AUCs greater than 0.7. When using SDS values, neither IGF-1 SDS nor IGFBP-3 SDS achieved an AUC greater than 0.7 in either sex. To improve the diagnostic performance of biomarkers for CPP, predictive models were developed using multivariable logistic regression, incorporating age, sex (coded as 1 for boys and 0 for girls), baseline LH, baseline FSH, IGF-1 SDS, and IGFBP-3 SDS. SDS values were separately calculated using the reference intervals from Roche and Jo et al., and the ROC curves of each model were compared.

The logistic regression equation incorporating age, sex, baseline LH, and baseline FSH can be expressed in an Excel-compatible formula as follows:

=1/(1 + EXP( − (−3.8582 + (0.1465 × [Age]) + (0.3318 × [Sex]) + (2.97 × [Baseline LH]) + (0.9595 × [Baseline FSH]))))

The logistic regression equation incorporating age, sex, baseline LH, baseline FSH, and IGF-1 SDS based on the Roche reference values can be expressed in an Excel-compatible formula as follows:

=1/(1 + EXP( − (−4.8635 + (0.2583 × [Age]) + (0.2239 × [Sex]) + (2.6901 × [Baseline LH] + (0.9459 × [Baseline FSH]) +( 0.2515 × [IGF-1 SDS-Roche])))))

The logistic regression equation incorporating age, sex, baseline LH, baseline FSH, and IGF-1 SDS from Jo et al.’s study can be expressed as an Excel-compatible formula as follows:

=1/(1 + EXP( − (−4.8408 + (0.2596 × [Age]) + (0.3306 × [Sex]) + (2.6986 × [Baseline LH]) + (0.9461 × [Baseline FSH]) + (0.2274 × [IGF-1 SDS-Jo et al.]))))

The logistic regression equation including IGF-1 SDS and IGFBP-3 SDS based on the Roche reference values can be expressed as an Excel-compatible formula as follows:

=1/(1 + EXP ( − (−5.443 + (0.326 × [Age]) + (0.69 × [Sex]) + (2.581 × [Baseline LH]) + (0.987 × [Baseline FSH]) + (0.444 × [IGF-1 SDS-Jo et al.]) +(−0.37 × [IGFBP-3 SDS-Jo et al.]))))

The logistic regression equation including IGF-1 SDS and IGFBP-3 SDS from Jo et al.’s study can be expressed as an Excel-compatible formula as follows:

=1/(1 + EXP ( − (−4.6697 + (0.239 × [Age]) + (0.3209 × [Sex]) + (2.536 × [Baseline LH]) + (0.988 × [Baseline FSH]) + (0.5077 × [IGF-1 SDS-Roche]) + (−0.4912 × [IGFBP-3 SDS-Roche]))))

The application of these predictive models demonstrated improved performance in both boys (highest AUC of 0.924) and girls (highest AUC of 0.800). There was no statistically significant difference in the AUC values derived from prediction modeling when comparing SDS-Roche and SDS-Jo et al.-based models (*p* > 0.05). The AUC values obtained from predictive modeling were significantly higher than those of baseline FSH alone (*p* < 0.05) in both sexes. In girls, the model incorporating the SDS values of IGF-1 and IGFBP-3 showed a statistically significant improvement in AUC compared to the model including only baseline LH and FSH, as well as the model including baseline LH, FSH, and IGF-1 SDS (*p* < 0.05). In boys, there were no significant differences in AUCs among the predictive models (*p* > 0.05).

**Figure 2:**
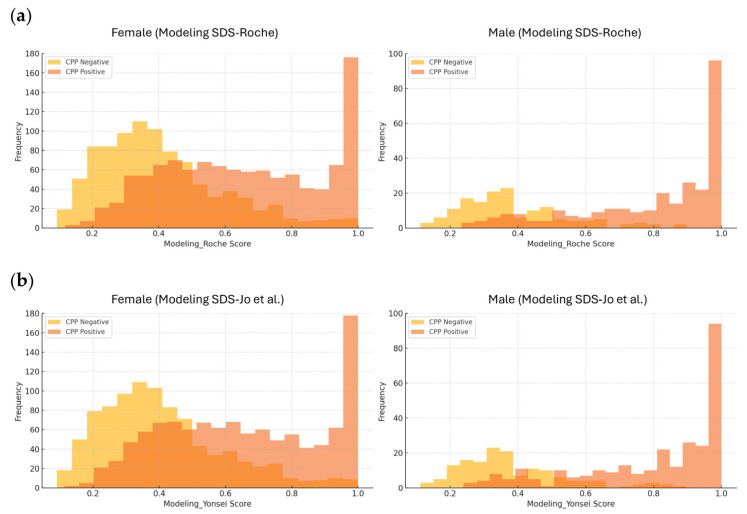
Histograms of predictive scores derived from models based on LH, FSH, IGF-1 SDS, and IGFBP-3 SDS. Histograms showing the distribution of predictive scores for central precocious puberty (CPP), stratified by sex and CPP status. (**a**) Predictive scores from the model using SDS values based on reference intervals provided by Roche. (**b**) Predictive scores from the model using SDS values based on reference intervals from the study by Jo et al. [10]. Different *y*-axis upper limits were applied for females and males, as the larger number of female patients caused the male bars to appear too small when a uniform scale (180) was used.

Figure 2 presents the predictive scores derived from the SDS-based model for individuals classified as positive or negative for CPP. Although there was some overlap between CPP and non-CPP individuals at lower predictive scores, a higher number of CPP cases was observed at higher predictive scores. Twenty patients (19 girls and one boy) without CPP but with high predictive scores (>0.9 in both models) exhibited elevated baseline LH levels (range: 0.4–4.5 IU/L) and elevated baseline FSH levels (range: 2.8–9.9 IU/L). In contrast, 13 patients (all girls) diagnosed with CPP had low predictive scores (<0.2 in both models) and showed decreased baseline LH levels (all < 0.3 IU/L) and decreased baseline FSH levels (range: 0.6–1.9 IU/L); 11 of them were under 7 years and 365 days of age.

## 4. Discussion

This study is the first to evaluate the diagnostic utility of baseline LH, baseline LH/FSH ratio, IGF-1, and IGFBP-3 measured using the Roche immunoassay platform in Korean children with suspected CPP. By applying multiple cutoff values—including the manufacturer-provided reference intervals and percentile thresholds derived from a Korean pediatric population—we aimed to assess the performance of these biomarkers as auxiliary tools for CPP diagnosis.

Although the GnRH stimulation test remains the definitive method for diagnosing CPP, its application in clinical practice is often limited by the need for multiple blood draws over an extended period—particularly challenging in pediatric settings [2,4]. Accordingly, there is growing interest in the development and validation of alternative or supplementary diagnostic strategies. In this context, it is important to recognize the inherent limitations of immunoassay-based hormone measurements [1]. Variability across assay platforms, differences in antibody specificity, and the lack of universal standardization contribute to inconsistencies in reference intervals and cutoff values [1,7,8,9,10,11]. Therefore, careful interpretation of results is required, especially when applying reference ranges across different populations. Population-specific validation of diagnostic thresholds is essential for accurate clinical decision-making and for minimizing the risk of misclassification [1,10].

Previous studies that proposed baseline LH cutoffs for CPP—such as 0.3 IU/L and 1.1 IU/L—were based on earlier generation assays including radioimmunoassays, which differ in analytical sensitivity and calibration from the current Roche platform [2]. In our study, baseline LH with the 0.3 IU/L threshold demonstrated higher AUCs in both sexes compared to the 1.1 IU/L threshold. In the present study, baseline FSH levels demonstrated the highest AUCs in both sexes when using raw values with sex-specific fixed cutoffs. However, the highest AUCs in both sexes—particularly in girls—were achieved through predictive modeling incorporating IGF-1 SDS and IGFBP-3 SDS values, suggesting that sex-specific considerations may be necessary when interpreting biomarker concentrations for CPP. The diagnostic performance and resulting prediction models based on SDS values derived from both the Roche reference data and the study by Jo et al. were comparable, supporting the practical feasibility of either approach as demonstrated in the present study. Although several studies have attempted to propose diagnostic cutoffs for IGF-1 and IGFBP-3 in CPP, most were limited by small sample sizes, inclusion of only girls within narrow age ranges, and lack of assay standardization [26,27,28,29]. Although a Korean study reported SDS values for IGF-1 and IGFBP-3 without specifying the analytical methods used, interpretation of these results should be approached with caution, as the Roche analytical methods for measuring IGF-1 and IGFBP-3 only became available for use in Korean clinical laboratories in October 2020 [10,30]. Previous studies conducted in Western populations have reported inconsistent results regarding the use of IGF-1 and IGFBP-3 for the diagnosis of CPP [26,27,28,29]. IGF-1 increases with rising sex hormones during puberty and precocious puberty, serving as a key marker of GH–IGF-1 axis activation and pubertal progression [1,26,27]. IGFBP-3 generally rises in parallel with IGF-1 but remains more stable, providing complementary information in the assessment of growth and pubertal status [1,26,27]. A prior study of girls with CPP and isolated premature adrenarche/precocious thelarche suggested diagnostic utility for IGF-1 SDS but not IGFBP-3 SDS; however, changes in assay methods, small sample size, and limited standardization underscore the need for further investigation [10,27]. A recent systematic review and meta-analysis suggested an association between CPP and elevated IGF-1 but not IGFBP-3; however, study heterogeneity and the lack of SDS adjustment emphasize the need for future studies incorporating SDS values to refine CPP diagnosis and management [28]. The findings of the present study are consistent with previous reports, demonstrating that IGF-1, but not IGFBP-3, shows significant differences relevant to the diagnosis of CPP. However, in our study, we developed a predictive model incorporating SDS values for IGF-1 and IGFBP-3, which resulted in improved AUCs. These findings highlight the potential clinical value of using interpretative reports that incorporate multiple hormonal markers to aid physicians in decision-making [18]. One strength of our study is that it provides calculation formulas that can be readily implemented using laboratory-generated data, enhancing its applicability in routine clinical laboratory settings.

In this context, the predictive performance for CPP could be further improved by incorporating additional clinical markers. Due to limitations in our dataset, clinical information such as body mass index (BMI), Tanner stage, and pelvic ultrasonographic findings was unavailable. Future studies that integrate such data may offer more refined and individualized prediction models for CPP diagnosis. Given that genetic polymorphisms may influence IGF-1, IGFBP-3, and susceptibility to CPP, future studies should incorporate these variants to better elucidate their clinical and biological impact [29]. Furthermore, beyond its role in supporting initial diagnosis, pre-treatment assessment of IGF-1 and IGFBP-3 may play an important role in evaluating growth potential and informing therapeutic decisions during treatment [1,31]. Follow-up studies exploring the clinical significance of IGF-1 and IGFBP-3 in both CPP diagnosis and treatment monitoring would be valuable.

Accurate SDS calculation requires robust reference data derived from a large number of healthy pediatric subjects. The importance of establishing pediatric reference intervals has been widely recognized, and several major initiatives have been launched globally to address this need. Notable examples include the Canadian Laboratory Initiative on Pediatric Reference Intervals (CALIPER), Australia’s Harmonizing Age Pathology Parameters in (HAPPI) Kids Study, the UK’s Pathology Harmony Group, the German Health Interview and Examination Survey for Children and Adolescents (KiGGS), and the Scandinavian Nordic Reference Intervals in CHILDren (NORICHILD) [31]. Additional efforts include the Pediatric Reference Intervals in China (PRINCE), the Pediatric Reference Range Initiative by the American Association for Clinical Chemistry (AACC, renamed the Association for Diagnostics & Laboratory Medicine [ADLM] in 2023), and the Children’s Health Improvement through Laboratory Diagnostics (CHILDx) initiative in the United States [31]. Establishing a nationwide, population-representative pediatric cohort in Korea would be a valuable step toward generating locally relevant pediatric reference intervals [31,32,33]. While the Korea National Health and Nutrition Examination Survey (KNHANES) allows for specimen collection from healthy individuals, its current sampling framework starts at age 10—limiting its applicability to CPP diagnosis, which typically occurs at younger ages [22,33]. Expanding such national efforts to include younger children would contribute to the establishment of more representative reference data, ultimately benefiting pediatric endocrine health on a population level [22,33].

In the present study, participants’ ages were close to the upper limit of the diagnostic criteria applied [3,4,5]. As this was a retrospective analysis of individuals who underwent GnRH stimulation testing, the observed age distribution may reflect patterns of hospital utilization (e.g., visits during elementary school years) as well as physicians’ decisions regarding the timing of the invasive GnRH stimulation test, which was performed with consent when clinical suspicion and signs became more evident at later ages [1,2,3,4,26]. Given the lack of detailed clinical information and the influence of age and developmental stage on hormone levels, the results of this study should be interpreted with caution and in the context of other findings, and future studies should further address how utilization patterns may affect the diagnostic implications of IGF-1 and IGFBP-3 [1,28].

## 5. Conclusions

This study is the first to assess the diagnostic utility of baseline LH, baseline FSH, IGF-1, and IGFBP-3 using the Roche-automated immunoassay in a Korean pediatric population with suspected CPP. By evaluating both manufacturer-based and population-derived cutoffs and comparing multiple biomarkers within the same cohort, it provides practical, assay-specific insights applicable to real-world clinical settings. The retrospective design and the study population—patients from local clinics and hospitals in Korea who underwent GnRH stimulation testing—may have introduced selection bias and limited the generalizability of the findings. Biomarkers were assessed at a single time point, and key clinical variables such as BMI, pubertal tempo, and longitudinal growth outcomes were unavailable. The absence of universally accepted, assay-specific pediatric reference ranges remains a key challenge [1]. Future prospective studies with larger, population-based cohorts are warranted to validate these findings. In particular, longitudinal designs that incorporate growth trajectories, bone age progression, and treatment outcomes would help clarify the clinical value of IGF-1 and IGFBP-3 as adjunctive diagnostic tools. Furthermore, efforts toward assay standardization and harmonization of reference intervals across populations and platforms are essential to improve the reliability and comparability of hormone testing in pediatric endocrinology [1,11,12,13].

## Figures and Tables

**Table 1:** Baseline characteristics of 2464 study subjects.

Characteristics	Median	Interquartile Range
Age (years)	8.3	7.7 to 8.9
Female (*n* = 2025)	8.1	7.6 to 8.8
Male (*n* = 439)	9.0	8.6 to 9.8
TSH (μIU/mL)	2.25	1.61 to 3.08
Baseline LH (IU/L)	0.21	0.21 to 0.40
Baseline FSH (IU/L)	2.00	1.40 to 2.80
Baseline LH/FSH ratio	0.14	0.10 to 0.21
IGF-1 (ng/mL)	201	160 to 244
IGF-BP3 (ng/mL)	4496	4066 to 4949
IGF-1/IGFBP-3 ratio	0.04	0.04 to 0.05
IGF-1 SDS-Roche	0.78	0.15 to 1.51
IGF-1 SDS-Jo et al.	0.61	−0.07 to 1.41
IGFBP-3 SDS-Roche	0.62	0.10 to 1.19
IGFBP-3 SDS-Jo et al.	0.54	−0.14 to 1.27
GnRH stimulation test		
Positive for CPP	1386	56.25%
Negative for CPP	1078	43.75%

Abbreviations: CPP, central precocious puberty; FSH, follicle-stimulating hormone; GnRH, gonadotropin-releasing hormone; IGF-1, Insulin-like growth factor-1; IGFBP-3, insulin-like growth factor binding protein-3; LH, luteinizing hormone; SDS, Standard Deviation Score; TSH, thyroid-stimulating hormone.

**Table 2:** Factors associated with central precocious puberty (CPP).

Characteristics	Female (*n* = 2025)			Male (*n* = 439)		
	Negative for CPP (*n* = 927)	Positive for CPP (*n* = 1098)	*p*-Value	Negative for CPP (*n* = 151)	Positive for CPP (*n* = 288)	*p*-Value
Age	7.98 (7.50 to 8.66)	8.24 (7.72 to 8.82)	<0.0001	8.86 (8.25 to 9.25)	9.38 (8.81 to 9.87)	<0.0001
TSH (μIU/mL)	2.24 (1.60 to 3.02)	2.22 (1.60 to 3.12)	0.8089	2.44 (1.75 to 3.17)	2.25 (1.65 to 3.04)	0.3922
Baseline LH (IU/L)	0.21 (0.21 to 0.21)	0.21 (0.21 to 0.50)	<0.0001	0.21 (0.21 to 0.21)	0.6 (0.21 to 1.10)	<0.0001
Baseline FSH (IU/L)	1.60 (1.20 to 2.10)	2.50 (1.90 to 3.30)	<0.0001	1.00 (0.73 to 1.40)	2.35 (1.80 to 3.20)	<0.0001
Baseline LH/FSH ratio	0.14 (0.10 to 0.19)	0.12 (0.090 to 0.19)	<0.0001	0.23 (0.15 to 0.30)	0.27 (0.15 to 0.47)	0.0183
IGF-1 (ng/mL)	189 (156 to 223)	216 (179 to 266)	<0.0001	173 (142 to 205)	200 (166 to 241)	<0.0001
IGFBP-3 (ng/mL)	4451 (4001 to 4910)	4500 (4070 to 4958)	0.1291	4443 (4132 to 4967)	4634 (4171 to 5062)	0.1832
IGF-1/IGFBP-3 ratio	0.04 (0.04 to 0.05)	0.05 (0.04 to 0.06)	<0.0001	0.04 (0.03 to 0.04)	0.04 (0.04 to 0.05)	<0.0001
IGF-1 SDS-Roche	0.56 (0.00 to 1.23)	0.95 (0.32 to 1.75)	<0.0001	0.72 (0.05 to 1.31)	0.91 (0.35 to 1.67)	0.0133
IGF-1 SDS-Jo et al.	0.44 (−0.17 to 1.16)	0.86 (0.17 to 1.71)	<0.0001	0.15 (−0.46 to 0.75)	0.35 (−0.14 to 1.08)	0.0100
IGFBP-3 SDS-Roche	0.58 (0.06 to 1.20)	0.60 (0.08 to 1.13)	0.8517	0.77 (0.23 to 1.36)	0.74 (0.21 to 1.28)	0.5194
IGFBP-3 SDS-Jo et al.	0.38 (−0.27 to 1.09)	0.43 (−0.20 to 1.09)	0.4306	1.17 (0.55 to 1.95)	1.35 (0.57 to 2.03)	0.3770

Data are presented as median (interquartile range), and *p*-values were calculated using the Mann–Whitney U test. Abbreviations: CPP, central precocious puberty; FSH, follicle-stimulating hormone; IGF-1, Insulin-like growth factor-1; IGFBP-3, insulin-like growth factor binding protein-3; LH, luteinizing hormone; SDS, Standard Deviation Score; TSH, thyroid-stimulating hormone.

**Table 3:** Baseline LH, baseline FSH, IGF-1, and IGFBP-3 Levels based on different cutoffs and upper reference limits for CPP diagnosis.

Categories	Female (*n* = 2025)							Male (*n* = 439)						
	Biomarker High		*p*-Value	Sen.	Spec.	PPV	NPV	Biomarker High		*p*-value	Sen.	Spec.	PPV	NPV
	No (*n*, %)	Yes (*n*, %)						No (*n*, %)	Yes (*n*, %)					
Baseline LH high (>0.3 IU/L)														
Negative for CPP	875 (94.4%)	52 (5.6%)	<0.01	35.7%	94.4%	88.3%	55.3%	141 (93.4%)	10 (6.6%)	<0.01	72.6%	93.4%	95.4%	64.1%
Positive for CPP	706 (64.3%)	392 (35.7%)						79 (27.4%)	209 (72.6%)					
Baseline LH high (>1.1 IU/L)														
Negative for CPP	925 (99.8%)	2 (0.2%)	<0.01	8.9%	99.8%	98.0%	48.1%	151 (100%)	0 (0.0%)	<0.01	24.3%	100.0%	100.0%	40.9%
Positive for CPP	1000 (91.1%)	98 (8.9%)						218 (75.7%)	70 (24.3%)					
Baseline FSH high (>1.8 IU/L for female, >1.6 IU/L for male)														
Negative for CPP	582 (62.8%)	345 (37.2%)	<0.01	76.4%	62.8%	70.9%	69.2%	129 (85.4%)	22 (14.6%)	<0.01	79.5%	85.4%	91.2%	68.6%
Positive for CPP	259 (23.6%)	839 (76.4%)						59 (20.5%)	229 (79.5%)					
IGF-1 high (>97.5th of Roche)														
Negative for CPP	868 (97.6%)	59 (6.4%)	<0.01	14.3%	93.6%	72.7%	48.0%	134 (88.7%)	17 (11.3%)	0.25	15.3%	88.7%	72.1%	35.5%
Positive for CPP	941 (85.7%)	157 (14.3%)						244 (84.7%)	44 (15.3%)					
IGF-1 high (>97.5th of Jo et al.)														
Negative for CPP	808 (87.2%)	119 (12.8%)	<0.01	21.1%	87.1%	66.1%	48.3%	140 (92.7%)	11 (7.3%)	0.24	10.8%	92.7%	73.8%	35.3%
Positive for CPP	866 (78.9%)	232 (21.1%)						257 (89.2%)	31 (10.8%)					
IGFBP-3 high (>97.5th of Roche)														
Negative for CPP	870 (93.9%)	57 (6.1%)	0.23	4.9%	93.9%	48.6%	45.5%	133 (88.1%)	18 (11.9%)	0.55	10.1%	88.1%	61.7%	33.9%
Positive for CPP	1044 (95.1%)	54 (4.9%)						259 (89.9%)	29 (10.1%)					
IGFBP-3 high (>97.5th of Jo et al.)														
Negative for CPP	847 (91.4%)	80 (8.6%)	0.96	8.6%	91.4%	54.0%	45.8%	133 (88.1%)	18 (11.9%)	0.86	12.5%	88.1%	66.7%	34.5%
Positive for CPP	1004 (91.4%)	94 (8.6%)						252 (87.5%)	36 (12.5%)					

Abbreviations: CPP, central precocious puberty; FSH, follicle-stimulating hormone; IGF-1, Insulin-like growth factor-1; IGFBP-3, insulin-like growth factor binding protein-3; LH, luteinizing hormone; NPV, negative predictive value; PPV, positive predictive value; Sen., sensitivity; Spec. specificity. This table presents 2 × 2 diagnostic performance outcomes for each biomarker evaluated in relation to CPP. The term ‘biomarker high’ refers to the application of a predefined cutoff value for the corresponding biomarker; for example, a baseline LH > 0.3 IU/L was applied for the first two corresponding rows. For each biomarker, diagnostic indices including *p*-value, sensitivity, specificity, PPV, and NPV are provided. The reference standard for defining CPP positivity or negativity was the result of the GnRH stimulation test.

**Table 4:** Summary of area under the curve (AUC) values from different receiver operating characteristic (ROC) curve analyses.

Variable	Female (*n* = 2025)		Male (*n* = 439)	
	AUC	95% CI	AUC	95% CI
Baseline LH	0.653	0.632 to 0.674	0.845	0.807 to 0.877
Baseline FSH	0.767	0.748 to 0.786	0.895	0.862 to 0.922
Baseline LH/FSH ratio	0.561	0.539 to 0.583	0.569	0.521 to 0.615
IGF-1	0.641	0.620 to 0.662	0.645	0.598 to 0.690
IGFBP-3	0.520	0.498 to 0.542	0.539	0.491 to 0.586
IGF-1/IGFBP-3 ratio	0.655	0.634 to 0.676	0.651	0.605 to 0.696
Based on baseline LH cutoff				
>0.3 IU/L	0.653	0.632 to 0.674	0.845	0.807 to 0.877
>1.1 IU/L	0.544	0.522 to 0.565	0.622	0.574 to 0.667
Based on Cutoff (>97.5th percentile)				
IGF-1 (Roche)	0.540	0.518 to 0.562	0.520	0.472 to 0.568
IGF-1 (Jo et al.)	0.541	0.519 to 0.563	0.517	0.470 to 0.565
IGFBP-3 (Roche)	0.506	0.484 to 0.528	0.509	0.461 to 0.557
IGFBP-3 (Jo et al.)	0.541	0.488 to 0.532	0.517	0.470 to 0.565
Based on SDS				
IGF-1 SDS-Roche	0.612	0.591 to 0.634	0.572	0.524 to 0.619
IGF-1 SDS-Jo et al.	0.610	0.588 to 0.631	0.575	0.527 to 0.621
IGFBP-3 SDS-Roche	0.502	0.480 to 0.524	0.519	0.471 to 0.566
IGFBP-3 SDS-Jo et al.	0.510	0.488 to 0.532	0.526	0.478 to 0.573
Based on prediction model–derived scores				
Model using LH and FSH	0.783	0.765 to 0.801	0.924	0.896 to 0.947
Model using LH, FSH, IGF-1 SDS-Roche	0.791	0.772 to 0.808	0.919	0.889 to 0.943
Model using LH, FSH, IGF-1 SDS-Jo et al.	0.790	0.772 to 0.808	0.920	0.889 to 0.943
Model using LH, FSH, IGF-1 SDS & IGFBP-3 SDS-Roche	0.800	0.782 to 0.818	0.919	0.889 to 0.943
Model using LH, FSH, IGF-1 SDS & IGFBP-3 SDS-Jo et al.	0.800	0.782 to 0.818	0.920	0.891 to 0.944

Abbreviations: AUC, area under the curve; CI, confidence interval; FSH, follicle-stimulating hormone; IGF-1, Insulin-like growth factor-1; IGFBP-3, insulin-like growth factor binding protein-3; LH, luteinizing hormone; SDS, Standard Deviation Score.

## Data Availability

The datasets generated and analyzed during the current study are available from the corresponding authors on reasonable request. The data are not publicly available due to privacy or ethical restrictions.

## Abbreviations

The following abbreviations are used in this manuscript:

AUC	area under the curve
CPP	central precocious puberty
FSH	follicle-stimulating hormone
GnRH	gonadotropin-releasing hormone
IGF-1	insulin-like growth factor-1
IGFBP-3	insulin-like growth factor binding protein-3
KNHANES	Korea National Health and Nutrition Examination Survey
LH	luteinizing hormone
ROC	receiver operating characteristic
SDS	Standard Deviation Score
TSH	thyroid-stimulating hormone
